# Deciphering the role of autophagy in the immunopathogenesis of inflammatory bowel disease

**DOI:** 10.3389/fphar.2022.1070184

**Published:** 2022-11-14

**Authors:** Yue Li, Helen Ka Wai Law

**Affiliations:** Department of Health Technology and Informatics, Faculty of Health and Social Science, The Hong Kong Polytechnic University, Hunghom, Hong Kong SAR, China

**Keywords:** inflammatory bowel disease, autophagy, immunity, pathology, immunopathogenesis

## Abstract

Inflammatory bowel disease (IBD) is a typical immune-mediated chronic inflammatory disorder. Following the industrialization and changes in lifestyle, the incidence of IBD in the world is rising, which makes health concerns and heavy burdens all over the world. However, the pathogenesis of IBD remains unclear, and the current understanding of the pathogenesis involves dysregulation of mucosal immunity, gut microbiome dysbiosis, and gut barrier defect based on genetic susceptibility and environmental triggers. In recent years, autophagy has emerged as a key mechanism in IBD development and progression because Genome-Wide Association Study revealed the complex interactions of autophagy in IBD, especially immunopathogenesis. Besides, autophagy markers are also suggested to be potential biomarkers and target treatment in IBD. This review summarizes the autophagy-related genes regulating immune response in IBD. Furthermore, we explore the evolving evidence that autophagy interacts with intestinal epithelial and immune cells to contribute to the inflammatory changes in IBD. Finally, we discuss how novel discovery could further advance our understanding of the role of autophagy and inform novel therapeutic strategies in IBD.

## Introduction

Inflammatory bowel disease (IBD) is a chronic, incurable, and debilitating non-specific inflammation, characterized by recurrent mucosal inflammation in the gastrointestinal tract. Different from other gastrointestinal inflammation, IBD has long-last effect including fibrosis, disability, and even cancer. IBD includes three major subtypes namely, Crohn’s disease (CD), ulcerative colitis (UC), and inflammatory bowel disease type unclassified (IBDU) (Chandradevan et al., 2018). CD always presents as discontinuous inflammation and ulceration in any region of the intestine from mouth to anus. In contrast, UC only involves the rectum and the colon with a continuous mucosal injury (Shivashankar and Lichtenstein, 2018). Besides, the difference in the affected locations, patients with CD always exhibited transmural inflammation with fissuring ulceration, and granulomas histologically, while UC presents cryptitis and crypt abscesses limited to the mucosa and submucosa (Guan, 2019).

IBD is an inflammatory disease associated with complex environmental and genetic factors. It is widely believed that IBD results from the combined effects of mucosal immunity dysregulation, gut microbiome dysbiosis, and gut barrier defect associated with genetic susceptibility and environmental triggers. The interaction of these factors leads to the complexity of the disease and the difficulty of treatment (de Souza et al., 2017). It is still unknown which factors initiate or inhibit the inflammation. The involvement of the innate immune system and the adaptive immune system in the progression of IBD is certain (Holleran et al., 2017). Moreover, the gut microbiota in IBD has a diminished diversity and is increasingly susceptible to colonization with pathogens or pathobionts (Ananthakrishnan et al., 2018). Genome-Wide Association Study (GWAS) suggests that endoplasmic reticulum stress, barrier integrity, innate immunity, autophagy, cytokine production, lymphocyte activation, the response to bacteria, and the JAK-STAT-pathway are related to IBD (Wang et al., 2019). At the same time, environmental factors greatly complicate the pathogenesis of IBD. Although IBD has complex pathogenesis, any pathogenic factor cannot be considered in isolation. Genetic susceptibility also requires the presence of specific disease-causing microorganisms or impaired immunity (Caruso et al., 2019). And the interaction between microbiota and mucosal immunity acts on intestinal inflammation (Chudnovskiy et al., 2016; Blander et al., 2017; Britton et al., 2019).

Intriguingly, IBD ultimately presents to be a chronic inflammation, directly caused by dysregulated immune responses. Hence, the exploration of immunopathogenesis is one of the most important therapeutic targets in IBD. Within the context of large-scale sequencing analysis, the identification of novel genes reiterates the central roles of innate and adaptive immune cells as well as autophagy in IBD pathogenesis (Sazonovs et al., 2022). Autophagy is implicated in diverse biological processes of IBD. As we will describe in this review, autophagy is the critical clue to draw together different pathogenesis, and demonstrate the link between immunity abnormalities and other pathogenic factors. We will focus on the immunity changes related to autophagy.

## The core autophagic machinery

Autophagy refers to a conserved cellular self-degradative pathway that involves the delivery of cytoplasmic organelles, proteins, and macromolecules to the lysosome and the recycling process (Parzych and Klionsky, 2014). There are three types of autophagy identified as microautophagy, macroautophagy, and chaperone-mediated autophagy. Macroautophagy is the major self-degradative pathway and in this proposal “autophagy” represents macroautophagy (Shao et al., 2016). Autophagy is a dynamic and complex process including autophagosome formation, selection of cargo, autophagolysosome formation, and final degradation (Ravanan et al., 2017). In general, conditions such as starvation, hypoxia, oxidative stress, and endoplasmic reticulum stress can activate the autophagy process.

Autophagy process includes the formation of phagophore, autophagosome, and degradation in the end. Phagophore is a double-membrane structure (Mizushima, 2018). During this period, the ULK1 complex (ULK1, ATG13, FIP200, and ATG101) is first initiated. Then phosphorylates PI3KC3 complex I (Class III PI3K, Beclin 1, ATG14, AMBRA1, and p115), which helps to activate PI3P production. PI3P then recruits WIPI2 and DFCP1 to the omegasome, where the transmembrane-bound ATG9 provides the double-membranes that form the phagophore (Levine and Kroemer, 2019). Elongation of the phagophore WIPI2 can bind ATG16L1 and recruit the ATG12∼ATG5-ATG16L1 complex, which will enhance the LC3 proteins and GABARAPs to membrane-resident phosphatidylethanolamine (PE) by ATG7 and ATG3 (Mizushima et al., 2011). In this process, LC3-I will be converted into LC3-II, the characteristic signature of autophagic membranes. With the elongation and sealing of autophagic membranes, autophagosome is formed (Dikic and Elazar, 2018). Finally, the autophagosome fused with the lysosome can degrade the autophagic cargo and recycle nutrients through receptors including CALCOCO2, SQSTM1, NBR1, and OPTN (Mizushima, 2018).

According to the research on the genes and genetic loci related to IBD, some pathways of autophagy are involved. Firstly, it was found that NOD2 recruited the autophagy protein ATG16L1 to the plasma membrane at the bacterial entry site. In IBD, mutant NOD2 failed to recruit ATG16L1 and impaired the process of autophagy (Cooney et al., 2010). Also, synonymous single nucleotide polymorphisms (SNP) in the auto-phagocytic gene ATG16L1 (such as T300A substitution), as well as an autophagy factor IRGM, are associated with increased risk for CD (Hampe et al., 2007). Study shows that the dysfunction of ATG16L1 in T cells in mice will lead to intestinal inflammation, abnormal Th (T helper) 2 responses, and a decrease in Foxp3+ regulatory T (Treg) cell numbers (Kabat et al., 2016a). Many IBD-associated genes are found to regulate multiple steps of autophagy. For example, NOD2 and LRRK2 participate in pathogen recognition, CALCOCO2/NDP52, and IRGM target to combine the bacteria. ATG16L1 and GPR65 are involved in autophagosome and autolysosome functions. ATG16L1, SMURF1, and PEX13 regulate inflammasome activation and cytokine production by mitophagy. ATG16L1, as the core autophagy protein, can also regulate the secretion of mucins and lysozyme in IBD (Lassen and Xavier, 2018).

## Autophagy and intestinal barrier function

The intestinal tract is a special immune organ. The intestinal barrier includes the mucus layer, intestinal epithelium, and gut-associated lymphoid tissue (GALT) (Stange and Schroeder, 2019). The first physical gut barrier isolates the microbiota and harmless food antigens from the gut mucosa is the mucus layer. The second gut barrier defenses against bacterial invasion are the intestinal epithelium, which consists of intestinal epithelial cells and other specialized epithelial cells such as goblet cells and absorptive enterocytes (Antoni et al., 2014). The third barrier, GALT, is comprised of the mesenteric lymph nodes, the Peyer’s patches, isolated lymphoid follicles, and colonic patches. The intestinal mucus layer, the epithelium, and the GALT are the site where immunological processes occur (Kabat et al., 2016b).

Functional studies emphasized the potential role played by dysfunctional autophagy in the antimicrobial response of the intestinal epithelial barrier (Foerster et al., 2022). Antimicrobial peptides (AMPs) are another significant component in the mucus layer. The functions of AMPs in IBD have aroused wide concern. The most important AMPs are the α-defensins (HD5 and HD6) and ß- defensins (HBD1, HBD2, and HBD3) produced by Paneth cells (Yu et al., 2020). It has been reported that HD5 was the candidate biomarker in Crohn’s colitis, involving in the colonic ectopy ileal metaplasia formation and disease development (Williams et al., 2017; Rana et al., 2021). Paneth cells are strongly associated with autophagy-related genetic variation in NOD2 and ATG16L1 (Stappenbeck and McGovern, 2017). A diminished expression of AMPs has been reported in chronic CD, especially in patients with NOD2 mutations. In response to pathogens, Paneth cells secrete abundant AMPs through the autophagy-related secretory pathway. Hence, the autophagy deficiency in the Paneth cells of the mice with the ATG16L1 mutation can increase the risk of CD (Bel et al., 2017). However, the colon contains few or no Paneth cells, so the mechanism of colonic AMPs remains unknown (Wehkamp and Stange, 2010). Besides, in *S. Typhimurium* infected-IEC mice model, *S. Typhimurium* can block the conventional secretion of lysozyme and increase the autophagy-dependent secretion of lysozyme, which means the autophagic defect in IEC will impair the antimicrobial function (Bel et al., 2017). This theory is compatible with studies showing that defects in ATG16L1 or IRGM contribute to the replication and survival of adherent-invasive *E. coli in vitro* (Lapaquette et al., 2010).

## Autophagy and innate immune system

Innate immune system maintains gut immune homeostasis by continuous regulation of the balance between proinflammation cytokines and antiinflammation cytokines through macrophages, dendritic cells (DCs), and some nonimmune cells (Park et al., 2017). This process is mediated by pattern-recognition receptors (PRRs), including toll-like receptors (TLRs) and NOD-like receptors (NLRs) that recognize pathogen-associated molecular patterns (PAMPs). Any disorders in different steps will cause inadequate immune response and inflammation and finally confer risk for IBD (Ahluwalia et al., 2018). In IBD patients, inflammation is promoted by inducing the production of pro-inflammatory cytokines, such as TNF, IL-1β, IL-6, and IL-18. While anti-inflammatory responses are promoted through the induction of IL-10 and IFN-α (Park et al., 2017).

### Macrophages

Macrophages are important innate immune cells, which can produce different cytokines, growth factors, and lipid mediators. Macrophages help to clear pathogens, bacterial wall components, and apoptotic cells (Hine and Loke, 2019). According to GWAS, monocyte-macrophage related genes are strongly-enriched in IBD, involving the adaptation of macrophages to the gut wall, their response to bacterial stimuli, and how their functions are integrated (Baillie et al., 2017). Intestinal macrophages play an important role in intestinal immune homeostasis by maintaining the balance between antigen tolerance and pathogen defense. Some studies show the potential function of macrophage differentiation in patients with IBD as the resolution of inflammation in IBD relies on the local recruitment of monocytes and accumulation of alternatively activated macrophages with pro-resolving capacity (Na et al., 2019). Different from the macrophage in other tissues, intestinal macrophages are highly specialized to be hyporesponsive to gut microbiota and negative in producing proinflammatory cytokines (Bain and Mowat, 2014). Tissue-resident tolerogenic macrophages mediate the secretion of regulatory cytokines IL-10, which maintains the survival of intestinal-resident Treg cells (Caer and Wick, 2020), and stop them from attacking the commensal bacteria. Human intestinal macrophages (CD45+HLA−DR+CD14+CD64+) can be divided into two subsets including monocyte-like cells (CD11c^high^CCR2+CX3CR1+, M1-like macrophage) and macrophage-like tissue-resident cells (CD11c−CCR2−CX3CR1−, M2-like macrophage). It has been reported that CD11c^high^ monocyte-like cells were increased in the inflamed colon in IBD (Bernardo et al., 2018). They are responsible for the overproduction of pro-inflammatory cytokines, such as IL-1β, IL-6, IL-23, IL-12, CCL11, and TNF-α (Caprara et al., 2020). Some studies also have shown that intestinal macrophages in CD patients always present abnormal morphological maturation and prolonged intracellular bacterial survival (Dige et al., 2016). Although intestinal M1-like macrophages increased and promoted inflammation during colitis, M2-like macrophages are also present to resolve inflammation (Pan et al., 2022). These findings suggested that enforcing the pro-resolving phenotype (M2-like macrophage) might represent a great potential for controlling the inflammation and prolonging remission (Hong et al., 2022). An interesting research demonstrated that intraperitoneal injection of bone-marrow derived M2 macrophages significantly reduced colitis symptoms (Ackermann et al., 2021; Honda et al., 2021).

With reference to autophagy, ATG16L1^T300A^ polymorphism can modulate TLR- and NLR-mediated signaling in IBD (Gao et al., 2022). This finding suggested that autophagy related to innate immunity was one of the potential mechanisms of IBD. Interestingly, it has been reported that mice with ATG16L1 defect can exacerbate dextran sulfate sodium (DSS) -induced colitis with an increased ratio of M2-like to M1-like macrophages, and pro-inflammatory cytokine production (Zhang et al., 2017). One of the most important functions of macrophage is bacterial clearance and it relies on autophagy. For example, the loss of PTPN2 in IBD patients’ macrophages will also reduce bacterial clearance resulting from autophagy defects (Spalinger et al., 2022). Wu’s study observed upregulation of NRBF2, a regulatory subunit of the ATG14-BECN1/Beclin 1-PIK3C3/VPS34 complex, which positively regulates autophagy, in the colon macrophages of UC patients (Wu et al., 2021). The MTMR3 risk allele is also found to enhance innate receptor-induced signaling and cytokines by decreasing autophagy in CD patients (Lahiri et al., 2015). On the other hand, another study reveals that adenosine monophosphate-activated protein kinase (AMPK)-induced autophagy could induce the anti-inflammatory response through intestinal macrophages and relieve DSS-induced colitis (Liu et al., 2020).

### Innate lymphocytes

Innate lymphocytes (ILCs) play an important role in the pathogenesis of IBD (Geremia and Arancibia-Carcamo, 2017). Although lacks the antigen-specific receptor (different from the adaptive immune cell), ILCs can rapidly respond to pathogens during the early immune period (Geremia and Arancibia-Carcamo, 2017). For the subtypes, ILC1s are mainly present in the upper gastrointestinal tract, while ILC2s are present in the entire intestine, and ILC3s number increases toward the colon (Kramer et al., 2017). ILC1s express Tbet and produce IFN-γ, whereas ILC2s express GATA3 and RORα and produce IL-5 and IL-13. ILC3 express RORγt and AHR and produce IL-22 and/or IL-17, which can be divided into two subgroups based on the expression of NKp44 (Peters et al., 2016). Marianne et al. reported that the frequency of NKp44 + ILC3 was decreased in inflamed tissue and was correlated with disease severity (Forkel et al., 2019). At the same time, ILC1 was increased in CD and ILC2 was increased in UC (Forkel et al., 2018). However, later studies have identified that IL-22 has dual functions in intestinal homeostasis maintenance and inflammatory induction (Eken et al., 2014; Bauche et al., 2018). Recently, a study using the TNBS-induced fibrosis mouse model presents that ablating the autophagy gene Atg7 increases the expression of IL-23, leading to increased expression of IL-22 and increased fibrosis, which is related to ILCs (Mathur et al., 2019).

### Dendritic cells

The DC population in the intestine present heterogeneous with different classical dendritic cells (cDCs) and plasmacytoid dendritic cells (pDCs) or tissue-resident DCs and blood-monocyte-derived DCs (Takagi et al., 2016). The basic function of DCs is antigen presentation and subsequent immune induction. However, DCs in the intestine play a unique role in inducing immune tolerance and maintaining homeostasis (Stagg, 2018). In general, pDCs secrete IFN-α in response to a viral infection, but intestinal pDCs induce immune tolerance instead of producing IFN-αs (Won et al., 2019). During homeostasis, intestinal DC can induce Treg cells, based on the common functions of distinct DC subsets. On the contrary, the imbalance of DCs subset contributes to IBD (de Souza and Fiocchi, 2016).

In the mice model, over-expression of LRRK2 in macrophage inhibits autophagy while the CD-associated risk variant in the LRRK2 gene leads to consequently excessive pro-inflammatory cytokine secretion. Moreover, LRRK2 inhibitors can decrease TNF production by mouse DCs and ameliorate DSS-induced colitis in mice models (Takagawa et al., 2018). Besides, some studies show that autophagy defect is involved in DC-T-cell interactions and DC-epithelial cell interactions (Wildenberg et al., 2012; Strisciuglio et al., 2013). They find that DC with autophagy defect displayed loss of filopodia, altered podosome distribution, increased membrane ruffling, and reduced migration (Wildenberg et al., 2017).

### Natural kill cells

Natural kill (NK) cells play an important role in the innate immune system and also play an essential role in linking innate and adaptive immunity. NK cells secrete IFN-γ, which induces the differentiation of CD4+ T cells to Th1 subsets (Schleinitz et al., 2010). Normally, NK cells kill the target cells through two major pathways. One is induced apoptosis of target cells by secreting cytoplasmic granule toxins such as perforins and granzymes. Another pathway involves caspase-dependent apoptosis by the death receptors in target cells (Martín-Fontecha et al., 2004). Different from NK cells from blood, intestinal NK cells resemble “helper” NK cells, which also have dual functions in promoting antipathogen responses and in the maintenance of intestinal homeostasis (Yadav et al., 2011). For example, a study identified the intestinal NK cells and found that the subset NKp46 + CD3 − CD127 + NK cells express RORγt, produce high levels of IL-22 but not IFN-γ and IL-17 (Cella et al., 2009).

Now, more and more evidence proves that the proportion and differentiation of NK cells are changed in IBD patients. IFN-γ-producing NK cells are increased in inflamed mucosa of CD patients, while IL-22-producing NK cells are decreased compared with those in UC patients and healthy controls (Takayama et al., 2010). However, the role of NK cells in the pathogenesis of IBD is still elusive. Some experimental evidence has preliminarily explored the function of autophagy and NK cells. First, autophagy is involved in the differentiation of long-life memory NK cells by ATG3-dependent mechanism or ATG-FOXO1 interaction (O'Sullivan et al., 2015; Huang et al., 2019). And the deletion of ATG5 causes mitochondrial injury and disturbs the development of NK cells (O'Sullivan et al., 2016). Autophagy may be critical to the maturation of NK cells and subsequently changes in IBD.

### Pyrin domain-containing 3 inflammasome

Inflammasome is also a member of the innate immune system and responsible for the activation of inflammatory responses [30]. It is a multi-protein oligomer including the Pyrin domain-containing 3 (NLRP3) protein, procaspase-1, and adapter protein apoptosis-associated speck-like protein (ASC) (Shao et al., 2019). So far, several kinds of inflammasomes have been described, including NLRP1, NLRP2, and NLRP3. Among them, the NLRP3 inflammasome is the most characterized one (Suárez and Buelvas, 2015). Except for the induction of adaptive immune responses and secretion of cytokines, intestinal subepithelial macrophages, DCs, and other inflammatory and immune cells can also form the NLRP3 inflammasome, leading to the activation of proinflammatory cytokines such as IL-1β and IL-18 (He et al., 2016; Groslambert and Py, 2018).

The NLRP3 inflammasome has been widely reported to be associated with the pathogenesis of IBD. the induction of NLRP3 inflammasome leads to the aggravation of IBD by increasing the secretions of IL-1β and IL-18. So far, autophagy is also found involved in the regulation of NLRP3 inflammasome activation and subsequent IBD inflammation. For example, Hou et al. found a negative regulator of NLRP3-mediated inflammation, namely coiled-coil domain containing protein 50 (CCDC50) (Hou et al., 2022). CCDC50 is a macroautophagy/autophagy cargo receptor to recognize NLRP3 and delivers it to phagophores for degradation (Lin et al., 2022). In the mice colitis model, CCDC50-knockout mice show more severe intestinal inflammation and elevated NLRP3 inflammasome (Hou et al., 2022).

## Autophagy and the adaptive immune system

### Autophagy and T cells

Gut homeostasis requires a balance between regulatory and effector T cells, and the loss of balance may result in the development of IBD (Ueno et al., 2018). Transplantation of naïve T cells into immunodeficient mice can induce IBD-like disease, suggesting the role of T cells in IBD (Kamanaka et al., 2011). Previous studies indicated that Th1-related cytokines (TNF, IFN-γ, IL-12) and Th17-associated cytokines (IL-17A, IL-21, IL-23) are increased in CD patients (Annunziato et al., 2007). Whereas Th2-associated cytokines such as IL-4 and IL-13 and Th17-associated cytokines are increased in UC (Xu et al., 2014). GWAS has shown that Th17 responses driven by IL23 contribute to IBD, and the loss-of-function mutations in IL23R present protection from IBD (Jostins et al., 2012).

Gut-resident FoxP3+ CD4+ Treg cells and Foxp3-IL10+ type1 Treg (Tr1) cells play a unique role in the suppression of immune responses against harmless dietary antigens and commensal microorganisms (Tanoue et al., 2016; Cook et al., 2019; Cosovanu and Neumann, 2020). The changes in the proportion of Th17 and Treg cells can alter the balance of gut immunity and induce colitis in mice (Britton et al., 2019). Research shows that Th17 is hyperactive in transdifferentiating into Treg, Th1, and Th22-like cells in response to different environmental conditions in IBD patients.

In addition, there is a new subset of helper T cells, Th9 cells, exposed by IL-4 and TGF-β and secondary activation of a complicated network of transcription factors such as interferon regulatory factor 4 (IRF4) and Smads (Shohan et al., 2018). A study in 2014 shows elevated Th9 cells, and overexpression of IL-9 has been demonstrated in UC. These Th9 cells may disrupt the epithelial barrier, impair tolerance, and lead to inflammation (Gerlach et al., 2014).

In the ATG16L1^T300A^ mice model, Th1 and Th17 cells are increased (Lavoie et al., 2019). Dengjel et al. (2005) reported that activation of autophagy promotes Treg cell survival and inhibits pro-inflammatory Th2 cell expansion. Autophagy inhibition disturbs the balance between different Th cell types in the intestine, which means autophagy has a direct influence on the proportion of different types of T cells. Autophagic defect T cells have decreased the proportion of CD4+ and CD8+ T cells and alteration of T cell proliferation. For example, in the research on mice with ATG16L1 defect in CD4+ T cells, intestine inflammation was induced, Treg cells were lost, and Th2 type response present abnormally against dietary and microbiota antigens (Kabat et al., 2016a). Moreover, according to the research on the outer membrane vesicles (OMVs) secreted by commensal bacterium *Bacteroides fragilis*, OMA can transfer CD4+ T cells to Foxp3+ Treg cells through an autophagy-dependent way (Shen et al., 2012).

### Autophagy and B cells

Immunoglobulin A (IgA), the main subclass of immunoglobin in the gut, is an important modulator of the gut microbiota (Okai et al., 2017). High-affinity IgA can protect the host against infection and low-affinity IgA mediates the tolerance of the commensal microbiota (Palm et al., 2014). Okai et al. (2016) reported that high-affinity IgA was involved in gut microbiota regulation and murine colitis prevention (Okai et al., 2016). IgG galactosylation, which is associated with IgG functions, presents a lower level in IBD patients than in healthy individuals (Simurina et al., 2018). Besides, increased antibody-secreting plasma cells and a change in the proportion of immunoglobulin can be observed in IBD patients (Uzzan et al., 2016; Pararasa et al., 2019). However, patients with IgA deficiency only present with asymptomatic or slightly symptomatic (Fadlallah et al., 2018).

Functional autophagy is also required for B cell activation and plasma cell terminal differentiation. Other than IgA,antigen-specific IgM and IgG responses were impaired in mice lacking B cell ATG5 and these mice were more susceptible to Heligmosomoides polygyrus infection and intestinal inflammation (Conway et al., 2013). However, the research on autophagy-related B cell changes is limited. The pathogenic potential of B cells and immunoglobulins in IBD still requires more exploration.

## Conclusion

Autophagy modulates the balance of proinflammation and anti-inflammation cytokines through different pathways and different innate immune cells and nonimmune cells. Autophagy promotes the processing of intracellular pathogens and contributes to MHC II restricted endogenous antigen presentation, regulates the proportion of different types of T cells, and affects B and T cell homeostasis. Almost all immunity changes are connected to autophagy. As discussed in this review, defective autophagy may amplify intestinal inflammation by influencing the status of cytokine, innate and adaptive immune cells in IBD. It is envisaged that effective autophagy can keep the gut tending to intestinal tolerance and immune tolerance homeostasis through multiple balances. Therefore, non-toxic, and specific regulation of autophagy is a potential target for IBD treatment, especially when the current treatment methods are mostly immune-related therapies.

## Glossary

IBD: inflammatory bowel disease
CD: Crohn’s disease
UC: ulcerative colitis
IBDU: inflammatory bowel disease type unclassified
GWAS: Genome-Wide Association Study
ULK1: Unc-51-like kinase 1
ATG: autophagy-related protein
FIP200: RB1-inducible coiled-coil protein 1
PI3KC3: class III PI3K
AMBRA1: activating molecule in Beclin 1-regulated autophagy protein 1
PI3P: phosphatidylinositol-3-phosphate
WIPIs: WD repeat domain phosphoinositide-interacting proteins
DFCP1: zinc-finger FYVE domain-containing protein 1
PE: phosphatidylethanolamine
LC3: microtubule-associated protein light chain 3
GABARAPs: γ-aminobutyric acid receptor-associated proteins
CALCOCO2: coiled-coil domain-containing protein 2
OPTN: optineurin
SNP: single nucleotide polymorphisms
Th: T helper
Foxp3: forkhead box P3
Treg: regulatory T
GALT: gut-associated lymphoid tissue
AMPs: antimicrobial peptides
HD: α-defensins
HBD: ß- defensins
IEC: intestinal epithelial cell
PRRs: pattern-recognition receptors
TLRs: toll-like receptors
NLRs: NOD-like receptors
PAMPs: pathogen-associated molecular patterns
TNF: tumor necrosis factor
IL: interleukin
IFN: interferon
CCL: chemokine (C-C motif) ligand
DSS: dextran sulfate sodium
AMPK: adenosine monophosphate -activated protein kinase
ILCs: innate lymphocytes
OMVs: outer membrane vesicles
DCs: dendritic cells
cDCs: classical dendritic cells
pDCs: plasmacytoid dendritic cells
TNBS: trinitro-benzene-sulfonic acid
NK cells: natural kill cells
NLRP3: Pyrin domain-containing 3
ASC: apoptosis-associated speck-like protein
CCDC50: coiled-coil domain containing protein 50
Tr1: type1 Treg
IRF4: interferon regulatory factor 4
Ig: immunoglobulin
MHC: major histocompatibility complex
